# The Development of an Automatic Rib Sequence Labeling System on Axial Computed Tomography Images with 3-Dimensional Region Growing

**DOI:** 10.3390/s22124530

**Published:** 2022-06-15

**Authors:** Yu Jin Seol, So Hyun Park, Young Jae Kim, Young-Taek Park, Hee Young Lee, Kwang Gi Kim

**Affiliations:** 1 Department of Biomedical Engineering, Gachon University, 191, Hambangmoe-ro, Yeonsu-gu, Incheon 21936, Korea; tjfwlgns0518@gmail.com; 2 Departments of Radiology, Gil Medical Center, College of Medicine, Gachon University, Incheon 21936, Korea; nnoleeter@gilhospital.com; 3 Department of Biomedical Engineering, College of Medicine, Gachon University, 38-13 Docjeom-ro 3 Beon-gil, Namdong-gu, Incheon 21565, Korea; youngjae@gachon.ac.kr; 4 HIRA Research Institute, Health Insurance Review & Assessment Service (HIRA), Wonju-si 26465, Korea; youngtaek.park@gmail.com; 5 Department of Health Sciences and Technology, Gachon Advanced Institute for Health Sciences and Technology (GAIHST), Gachon University, Seongnam-si 13120, Korea

**Keywords:** artificial intelligence, image processing, three-dimensional region growing, ribs

## Abstract

This paper proposes a development of automatic rib sequence labeling systems on chest computed tomography (CT) images with two suggested methods and three-dimensional (3D) region growing. In clinical practice, radiologists usually define anatomical terms of location depending on the rib’s number. Thus, with the manual process of labeling 12 pairs of ribs and counting their sequence, it is necessary to refer to the annotations every time the radiologists read chest CT. However, the process is tedious, repetitive, and time-consuming as the demand for chest CT-based medical readings has increased. To handle the task efficiently, we proposed an automatic rib sequence labeling system and implemented comparison analysis on two methods. With 50 collected chest CT images, we implemented intensity-based image processing (IIP) and a convolutional neural network (CNN) for rib segmentation on this system. Additionally, three-dimensional (3D) region growing was used to classify each rib’s label and put in a sequence label. The IIP-based method reported a 92.0% and the CNN-based method reported a 98.0% success rate, which is the rate of labeling appropriate rib sequences over whole pairs (1st to 12th) for all slices. We hope for the applicability thereof in clinical diagnostic environments by this method-efficient automatic rib sequence labeling system.

## 1. Introduction

The burden on medical imaging-based diagnosis has been increased gradually with the demand for radiologic scanning [1,2]. As the demands for computed tomography (CT) imaging and reading have been increased, it is noted that research to reduce the burden on the radiologists while they read and improve the diagnostic process using an automatic system such as computer-aided diagnosis (CAD) [3,4]. The chest CT-based medical is usually read to diagnose diseases occurring in abdominal and design thoracic surgery, as it is fast and provides anatomical information about general tissues of the upper body [5,6]. Accordingly, research on CAD with an automatic system to aid image reading and diagnosis on chest CT slices has been conducted and published in several fields.

In clinical applications, radiologists check the image with their naked eyes repeatedly and make a diagnosis decision to increase the reliability of the reading results. Additionally, the read CT datasets are shared among readers with reference to the location and size of the interested anatomical tissues. For these reasons, radiologists must recheck to recognize the referred information and diagnose diseases using coordinates they predesignated within the CT. In the process of chest CT reading, the readers intuitively use indexes that can indicate the states of tissues, which is usually referred to by the ribs’ order. CT images can show bones clearly compared to other tissues, and in the case of ribs, they surround the thoracic cavity covering most of the body’s internal organs and become coordinates of the location of the region of interest throughout the upper body [7,8]. Accordingly, counting ribs from 1 to 12 and manually labeling the sequence of ribs has been an essential step whenever radiologists read chest CT to refer to the anatomical location. However, manually labeling ribs’ sequence at every reading is repetitive and time-consuming work even in emergency situations [9,10]. The development of an automatic rib sequence labeling system can reduce the burden on radiologists and improve the diagnostic environment using chest CT by annotations of sequence that can be displayed simultaneously with data input on the image slice of the CT. 

Previously, several studies on the segmentation of ribs and labeling their sequence have been published [11,12,13]. Staal et al. [14] published a labeling system by multiple deep learning networks on a 3D reconstructed model using chest CT. It achieved detailed labeling of rib structures using multiple networks of 3D classification and segmentation stage. In addition, Wessle et al. [15] proposed an automatic rib sequence labeling system for X-ray images, which annotates all the ribs’ labels on an X-ray slice. Prior studies used 3D deep learning networks in multiple numbers to label interesting ribs, which can cause overfitting when using a small number of medical imaging data. Image reformation is also needed to show labels in multiplanar reformation (MPR) views used by radiologists. In addition, detecting each rib using only deep learning networks requires sufficient training data of ribs in each sequence.

In this study, to label the ribs’ sequence, we proposed two methods, an intensity-based image processing (IIP) and a convolutional neural network (CNN)-based deep learning with 3D region growing. The methods can examine the ribs’ region’s 3D context and labeling in order without rendering and multinetworks. By comparison analysis between the two methods to segment ribs to label, we aimed to develop an optimal algorithm and achieve more significant results.

## 2. Materials and Methods

**Figure 1:**
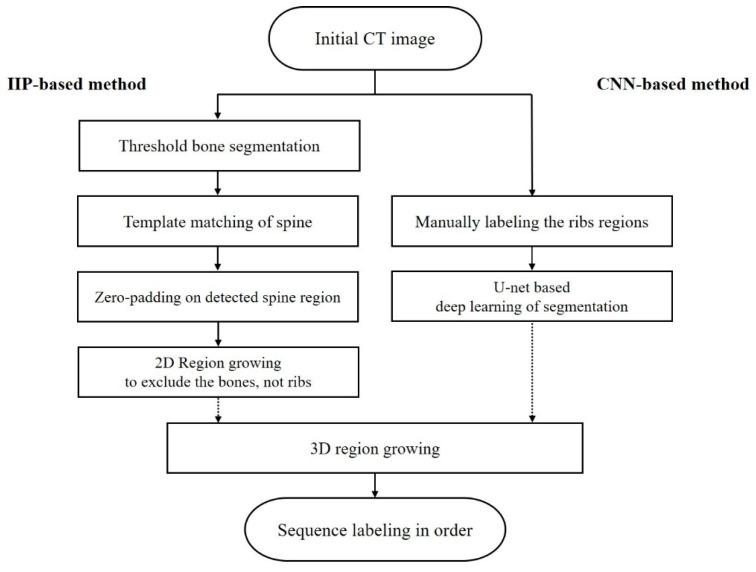
Flowchart of rib sequence labeling processes using IIP-based method and CNN-based method; (IIP—intensity-based image processing; CNN—convolutional neural network).

Rib regions to be labeled were segmented by two methods and the performances were compared: IIP- and CNN-based methods. For the IIP-based method, arithmetic statistics to distinguish between regions of interesting ribs and outliers were used in the process. The method can prevent a potential overfitting caused by limited medical imaging data and reduce overall development time, as a manual process of labeling the region of interest is not needed for training [16,17]. In contrast, for the CNN-based method, the semiautomatic ground truth was manually verified by trained readers, and U-net was used to create processed masks of the rib region to be labeled [12,18,19]. This U-net is a CNN that was developed for biomedical image segmentation [20,21]. The network has demonstrated high performance on computer-aided diagnosis in several prior studies related to bones, and it reduces more outliers because it is not a standardized process [22,23,24]. The created masks including only rib regions by these two methods were examined to label the 12 pairs of ribs using 3D region growing. We quantitatively investigated the performance of the systems from each technique based on the success rate, which is the rate of indexing appropriate rib sequences over whole pairs (1st to 12th) for 50 input datasets. Figure 1 shows a flowchart of the rib sequence labeling process. (Figure 1) 

### 2.1. Ethics Statement

This study was approved by the Institutional Review Boards of Gil Medical Center (GBIRB 2021-253). Informed consent was obtained from all the patients at the institution. All the experiments were performed with the relevant guidelines and regulations in accordance with the Declaration of Helsinki.

### 2.2. Data

Fifty patients who underwent chest CT in Gil Medical Center between January 2021 and March 2021 were randomly selected. Acquisition of all CT images was performed on a 256-channel CT (SIEMENS), which included axial reconstruction. The scans were reconstructed with section thickness less than or equal to 3 mm. The phantom of body was scanned with tube voltage of 120 kVp, while the CT X-ray tube current was varied with values ranging from 400 mA to 600 mA. Additionally, they were standard-dose images consisting of raw formats of the soft kernel, with dimensions of 512 × 512 pixels. Moreover, the tube potentials of scanned images were the same as 120 kV. The chest CT images of the 50 patients comprised 5250 sheets, each of which included the entire upper body. The range of pixel spacing for the collected data was 0.617 mm to 1.067 mm, with a mean of 0.798 mm and a standard deviation of 0.069. To collect the same number of sheets per patient, 50 CT sets were reformatted to unified pixel spacing of 0.798 mm.

Therefore, to apply proper image processing and to verify the performance of rib sequence indexing on CT images under the same conditions, the window widths and level settings of each image could affect image construction, they were unified to 400 HU and 40 HU, respectively, emphasizing the bones on the images.

The IIP-based method was constructed based on the collected 50 chest CT datasets, and 5250 slices were used to validate the method’s rib sequence labeling performance. For the CNN-based method, the 5250 collected CT images were divided into 32 patient cases including 3360 slices as the training data, 8 cases including 840 slices as the validation data used during training, and 10 cases including 1050 slices as the test data for the system. All collected data were evaluated through five-fold cross-validations.

### 2.3. Experiment Environment

Image preprocessing, IIP-based methods, and 3D region growing-based labeling were performed and the final system was designed to a graphical user interface (GUI) using C++ with Microsoft Visual Studio (Ver. 2010; Microsoft, Redmond, WA, USA). Furthermore, CNN-based methods were performed using Python 3.6.10 and the Keras 2.3.1 framework, using a system with four NVIDIA (NVIDIA, Santa Clara, CA, USA) RTX 2080 Ti GPUs, 128 GB RAM, ST2000DM008-2FR102, and WDC WDS500G2B0C-00PXH0 of storage.

### 2.4. Processing to Generate Masks of Interesting Rib Regions

#### 2.4.1. IIP-Based Method

In the IIP-based method, only IIP was used to separate the rib regions from other regions to label the sequence, which prevented the overfitting of the experimental data with faster data processing in limited data. To implement a system that labeled only ribs in order using the IIP-based method, nonrib outliers had to be excluded from the mask.

The original image datasets were converted into a series of binary images by a general threshold, retaining only the bones in the images; the threshold was above the average of the entire pixel range in the image of 140. Excluding regions with pixel values of 255, the other pixels were filled with zeros.

In binary images from chest CT, it was necessary to remove the vertebrae and sternum if they were in the images. They connect all the ribs from the front and back plates of the upper body. This resulted in 12 pairs of ribs being considered as a single whole bone during 3D region growing. Therefore, this study sought to remove the vertebrae and sternum from the mask through template matching to overcome the limitations of general image processing. Template matching is a method that detects the most appropriate area regarding similarity to predefined templates [25,26]. Here, template matching was used, which is based on equation (1), i.e., the sum of absolute differences (SAD) [27]. Template matching by SAD is a technique for extracting regions with the minimal sum of absolute values of pixel-to-pixel errors for the same coordinates (i.e., [*u*, *v*]) between consecutive pixels of the target input image (*I*_1_) and predefined templates (*I*_2_) [28,29]. We collected 15 cropped images indicating thoracic vertebrae with varied shapes, which were extracted in the chest CT images used as the predefined templates. Then, zero padding was applied based on the coordinates of the detected vertebral region by template matching. The zero-padding erased the vertebral and sternum regions facing each other to leave only rib regions on the created mask.

(1) $$\mathrm{SAD}=\underset{\left(u,v\right)\in I}{\sum }\left|I_{1}\left[u,v\right]-I_{2}\left[u,v\right]\right|$$

Subsequently, morphological computation erased the scanned bed and the remaining anomalies to reduce noises that can interfere with appropriate labeling. Additionally, for each image slice, a two-dimensional (2D) region growing was used to exclude bones that were not ribs. The 2D region growing used here began with a seed pixel and inspected the other four-connected neighborhood pixels around it, including a similar pixel for a specific criterion [30,31]. This process examined all adjacent pixels and continued until there were no additional pixels to be included, resulting in large coverage areas. To exploit it, we used seed pixels located across all regions with pixel values of 255 on each image slice in order, which indicated bones. From the pixel pointing to the image coordinate (0, 0) to the pixel pointing to the image coordinate (512, 512), the first point with a pixel value of 255 was selected as a seed pixel in each region, and all four-connected neighborhood pixels with a pixel value of 255 were included in the area. 

Thus, each region was labeled as an object in the mask, and the size of the region was determined by the number of pixels contained in the region. To leave only the area that indicated entirely the ribs in each slice mask, an object was excluded if it was not in contact with the vertebral region obtained from template matching at the first time of appearance on the slice. 

**Figure 2:**
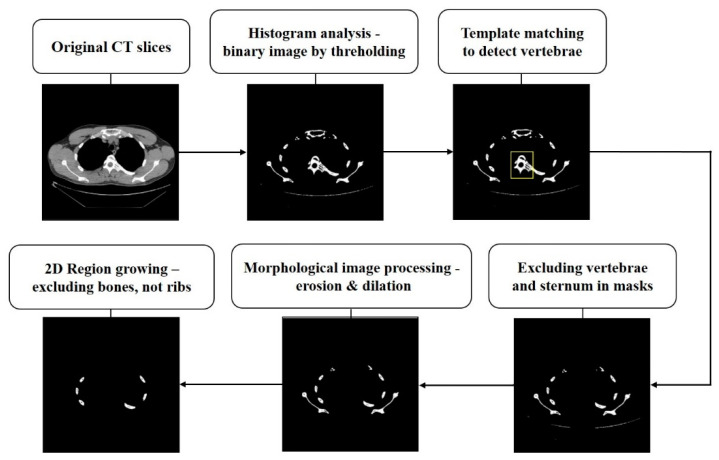
Results of image processing in the intensity-based image processing (IIP)-based method.

Figure 2 shows an example of the image preprocessing techniques used in the IIP-based method and the foreground. (Figure 2)

#### 2.4.2. CNN-Based Method

**Figure 3:**
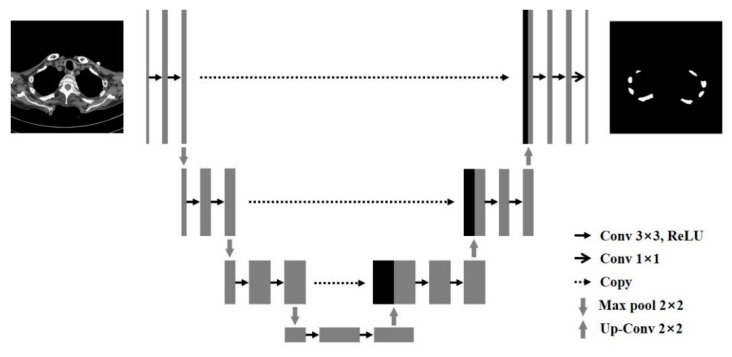
The architecture of U-net.

U-net was used as the network for the deep learning-based segmentation of the rib regions, which was based on a CNN. (Figure 3) U-net is a U-shaped structure that allows the encoding and decoding phases to be reused through skip connections between stages and combines the location information of down-sampling paths with the context information of up-sampling [28,32]. Thus, the segmentation network with U-net was advantageous in that it could accurately separate the regions of interest with few training images, resulting in high-quality medical images [33]. The learning environment was fixed with batch sizes of 8 and 300 epochs, and early stopping algorithms were used to prevent overfitting. In addition, five-fold cross-validation was used to verify the learning performance, which means that each learning consisted of 40 different cases to train and 10 cases to test for each training. It can use all datasets for training and testing and verify if it is overfitting. Additionally, all the data used in the CNN-based method were manually labeled and reviewed by trained readers. Two professional radiologists who participated as authors have drawn ground-truth and reviewed the data.

In the CNN-based method, the segmentation results of U-net are closely related to the results of labeling the rib sequence. Therefore, the trained models were evaluated by a quantitative evaluation and five-fold cross-validation.

### 2.5. 3D-Region Growing for Sequence Labeling

**Figure 4:**
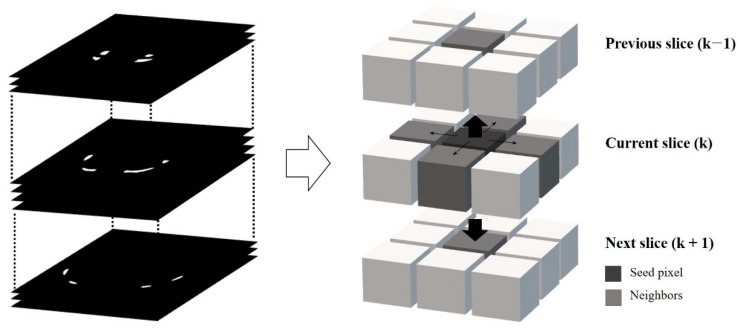
The principle of 3D region growing using 6-neighborhood.

Region growing is a method of segmentation that examines the pixel values which are adjacent to selected pixels as seed pixels in the input image. It includes the pixels whose values meet a predesignated criterion as general data clustering, and continues the examination until there are no more pixels to add [30,34]. Particularly in this study, 3D region growing was used to classify each rib in the 3D CT dataset. A 3D region growing examined near pixels in the x-, y-, and z-axis of the seed pixels (i.e., six-connected neighborhood) and included the pixels within a predetermined category which is considered to be one cluster. It can objectify pixel clusters referring to 12 pairs of ribs [35,36]. In several recent studies have been published using 3D region growing method to segment tumors and nodules in limited medical images according to the 3D imaging system in the medical field without extra training, which achieved significant performance in clinical decision [37,38]. In this study, it was designed that if the examined pixel value was 255 and it was adjacent to the seed pixel indicating the ribs, the pixel was included in the same index of the label. Figure 4 shows the processes of the overall 3D region growth by analyzing the adjacent pixel (Figure 4). 

When using a full set of chest CT images as input, beginning the examination from the first image slice, a total of 12 pairs of labels were provided to the object that appeared from the first slice in order. A total of 24 labels pointing to both ribs were provided to each region from the seed pixels of the 12 rib pairs, and the automatic sequence labeling system presented here was designed to present annotations simultaneously of only 12 ribs on the left side to improve the readability of chest CT. 

### 2.6. Statistical Analysis-Based Performance Evaluation 

The CNN-based method was implemented by means of U-net to segment the regions of the ribs of interest, and its performance was evaluated quantitatively using the dice similarity coefficient (DSC), precision, recall, and accuracy, defined in Equations (1)–(4). Through model learning, the positions of the segmented rib region were compared pixel by pixel, yielding true-positive (TP), false-positive (FP), true-negative (TN), and false-negative (FN) values, which were then used to calculate the aforementioned metrics. In this case, positive means pixels included in the rib regions, while negative means pixels of regions other than ribs.

(2) $$\mathrm{DSC}=2\frac{\mathrm{TP}}{\left(\mathrm{TP}+\mathrm{FP}\right)+\left(\mathrm{TP}+\mathrm{FN}\right)}$$

(3) $$\mathrm{Precision}=\frac{\mathrm{TP}}{\mathrm{TP}+\mathrm{FP}}$$

(4) $$\mathrm{Recall}=\frac{\mathrm{TP}}{\mathrm{TP}+\mathrm{FN}}$$

Additionally, for the performance evaluation of an automatic rib sequence labeling system on chest CT, the success rate was calculated. The success rate is the rate of cases labeling all 12 pairs of ribs in a complete sequence for 50 input datasets from 50 patients. The success rate of the overall labeling process using the IIP-based and CNN-based methods was evaluated based on the number of successful patient cases (the number of successful cases/total number of patient cases).

## 3. Results

Evaluating the performance of the segmentation network by U-net with the test data, the trained model achieved the performance of a DSC of 0.892, a precision of 0.906, recall of 0.920, specificity of 0.983, and accuracy of 0.979 for each mean value of the five-fold cross-validation. In addition, Table 1 presents the minimum and maximum values with 95% confidence interval (CI) of network performances achieved by five-fold cross-validation (Table 1). A 95% CI means a range of values that has 95% confident contains the true mean of the population. 

Consequently, to assess the performance of each system’s algorithm, 50 cases of upper-body CT with all pairs of ribs were used. The automatic rib segmentation and sequence labeling system using only IIP achieved a labeling success rate of approximately 92.0%. A deep learning network was applied to the system using CNN-based segmentation training, which obtained a labeling success rate of 98.0% for 50 cases. Table 2 compares the sequence labeling systems based on the IIP-based and CNN-based methods in terms of their successful labeling rates of five-fold cross-validation sets. In addition, the sequence labeling system recorded about 2 seconds and 14 seconds in an IIP-based method and a CNN-based method, respectively, each using a single GPU from input original CT dataset. This prevents repetitive time-consuming work and allows readers to efficiently handle medical image reading time with an automated system.

**Figure 5:**
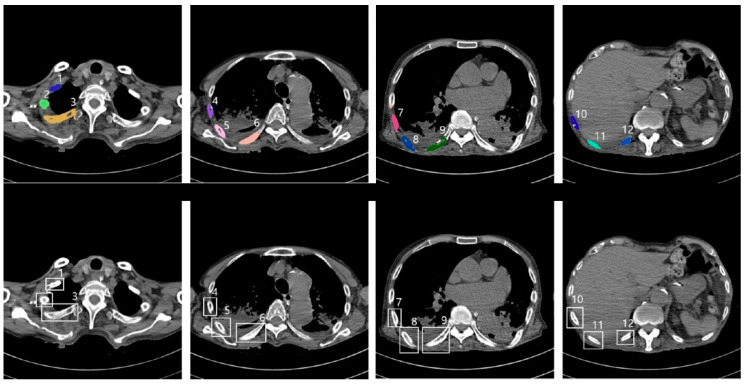
Results of sequence labeling; annotation for sequence labels with coloring (**top**) and boxes (**bottom**), including nearby numbers (1–12) labels indicating the order of the ribs from the top of the upper body (CNN, convolutional neural network).

**Figure 6:**
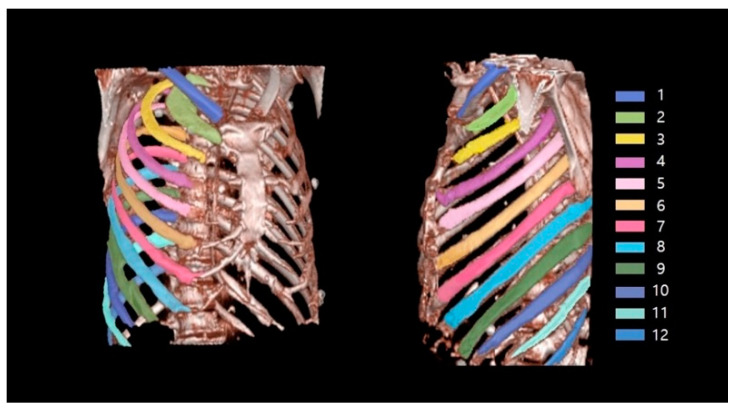
Verified results of sequence labeling on 3D-rendering rib models with colored annotations.

Figure 5 shows an example of successful automatic rib segmentation and sequence labeling (Figure 5). Intuitive results are displayed via colored labels and boxes in Figure 5. In addition, the result of sequence labeling is reconstructed on 3D rendering models with color annotations to verify the performance of the developed system (Figure 6).

## 4. Discussion

**Figure 7:**
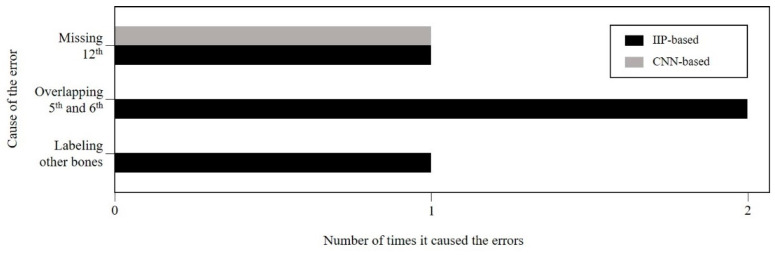
Cause of errors in intensity-based image processing (IIP)-based and convolutional neural network (CNN)-based methods.

This study compared the quantitative results of both methods to evaluate their respective sequence labeling performance. Both the IIP-based method and CNN-based method achieved high performance on sequence labeling tasks. The CNN-based method particularly can label the series of ribs sequence with one error. When we showed the performance of the IIP-based and CNN-based automatic rib sequence labeling systems, they are both expected to be efficiently used in medical imaging environments. However, in several cases, there were errors of the ribs’ labels that need improvement. The errors were included in the case of overlapped or missed, resulting in incorrectly ordered rib indexes. Figure 7 presents the cause of the error and the number of times it occurred in the IIP-based and CNN-based methods (Figure 7). Through the comparative analysis of the results, it can be distinguished which common errors occur for the system.

According to the reported results, there were errors in four cases in the IIP-based method and errors in only one case in the CNN-based method. Both methods failed to correctly label the 12th rib in one case each. In the case of the CNN-based method, it is the only cause of an error. The error was caused as the 12th rib was too small and can be seen dimly in the CT images depending on the patient taken. From the perspective of the radiologist, the 11th and 12th ribs are thinner and shorter than others anatomically. Depending on the patient’s case, it is rarely seen in CT images, and it is defined as having 11 pairs of ribs. therefore, in the future, we plan to collect more unusual data like these. In this experiment, the case of missing the 12th rib is the same case, which shows the rib area in only one slice connected to the spine. It was determined that when the region of spine was zero-padded with template matching and implemented morphological image preprocessing, the part of the small 12th rib’s area was erased [39]. To improve this, it is planned to apply a zero-padding to only pixels of template-matched regions and adjust the processing pixel parameter in morphological image preprocessing. 

Additionally, in the case of the IIP-based method, there were more errors including two cases in which the 5th and 6th ribs overlapped, and one case in which bones other than the ribs were misclassified as ribs and labeled. The errored cases of overlapping 5th and 6th caused the most errors in the same method. Anatomically, ribs are classified as true ribs (1st–7th), false ribs (8th–10th), and floating ribs (11th and 12th), and each type of rib is connected with the sternum relatively. The true ribs are located at the top of the rib cage and morphologically long. The 5th, 6th, and 7th ribs in the true ribs converge, attaching roughly to the same sternum site anatomically [40]. This caused on the axial CT image that the rib regions were overlapped with a transverse axis, which resulted in the same label. Therefore, the system will be developed in future work to label the more lateral regions of ribs, which appear to be separated a little further from the sternum. 

Lastly, several bones in contact with ribs, such as shoulder bones and clavicle, had errors in the process as they were referred to as a single bone in the IIP-based method. In this study of IIP-based method, there is an exceptional case of bones in a size and position similar to ribs, which were mistaken for ribs and could not be excluded from the image while the method excluded other bones using the 2D region growing method. However, these could be excluded through the CNN-based method. Therefore, in future studies, we plan to further prevent errors of exceptional cases and improve the performance of the system by including various rib cases in training in CNN-based methods.

## 5. Conclusions

This study applied IIP-based and CNN-based methods to develop an optimal automatic rib sequence labeling system on chest CT images. Both methods resulted in high performance values of labeling using a single network and simple image processing of 3D examination without rendering and reconstruction. In particular, the CNN-based method could segment rib regions and label the sequence with higher performance. A deep learning network of the CNN-based method are planned to be generalized through reinforcement learning to reduce analyzed errors, which is closely related to the results of accurate labeling. In addition, a CNN-based method will be trained with fractured and deformed cases to increase the versatility of the system in an actual clinical environment. These sequence labels from this proposed automatic system will be designed to show in the GUI of the medical image reading process through future research. Furthermore, by providing this system in medical image sharing networks such as PACS, we aim to further increase the utilization of ordered images and improve the reading environment. Moreover, We plan to investigate the satisfaction of professional radiologists at various levels after using it to compare with the manual methodology performed by radiologists in clinical practice.

## Figures and Tables

**Table 1:** Mean values of U-net performance in reconstructing the rib regions of interest.

	U-Net (CNN)				
	Recall (%)	Precision (%)	Specificity (%)	Accuracy (%)	DSC
Average	91.99	90.61	98.33	97.91	0.89
(95% CI)	(90.83–93.15)	(89.26–91.96)	(97.87–98.79)	(97.42–98.40)	(0.87–0.91)
Min	90.23	88.91	97.88	97.44	0.87
Max	93.31	91.87	98.91	98.82	0.92

**Table 2:** Comparison of the sequence labeling systems based on IIP and CNN in terms of successful labeling rate; (*p*-value, a probability value).

	Labeling		*p*-Value
	No. (Out of 50 cases in total) (Success cases Nr./Total cases Nr.)	Successful sequence labeling rate on all ribs (%) (95% CI)	*p* < 0.05
IIP-based	46/50	92.0% (85.4–98.6%)	
CNN-based	49/50	98.0% (94.5–100.0%)	

## Data Availability

The dataset used and analyzed here is available from the corresponding author upon reasonable request.

## Abbreviations

Variables and abbreviations written in this paper.

CT	computed tomography
IIP	intensity-based image processing
CNN	convolutional neural network
3D	3-dimensional
DSC	dice similarity coefficient
SAD	the sum of absolute differences
TP	true-positive
FP	false-positive
TN	true-negative
FN	false-negative
CI	confidence interval
